# Addressing racial/ethnic inequities in vaccine hesitancy and uptake: lessons learned from the California alliance against COVID-19

**DOI:** 10.1007/s10865-022-00284-8

**Published:** 2022-01-22

**Authors:** Mona AuYoung, Patricia Rodriguez Espinosa, Wei-ting Chen, Preeti Juturu, Maria-Elena De Trinidad Young, Alejandra Casillas, Paris Adkins-Jackson, Suellen Hopfer, Ed Kissam, Audrey Kawaiopua Alo, Roberto A. Vargas, Arleen F. Brown, And the STOP COVID-19 C. A. Communications Working Group

**Affiliations:** 1 Scripps Health, San Diego, CA USA; 2 Office of Community Engagement, Stanford University School of Medicine, Palo Alto, CA USA; 3 Department of Epidemiology and Population Health, Stanford University School of Medicine, Palo Alto, CA USA; 4 Center for Health Disparities Research, University of California at Riverside, Riverside, CA USA; 5 Department of Public Health, University of California at Merced, Merced, CA USA; 6 Division of General Internal Medicine and Health Services Research, Department of Medicine, UCLA David Geffen School of Medicine, Los Angeles, CA USA; 7 Division of Neurology, Massachusetts General Hospital, Boston, MA USA; 8 Harvard Medical School, Boston, MA USA; 9 Department of Health, Program in Public Health, University of California at Irvine, Society, & Behavior, Irvine, CA USA; 10 Center for Reducing Health Disparities, University of California, Davis, Davis, CA USA; 11 Werner Kohnstamm Family Giving Fund, Sacramento, CA USA; 12 Pacific Islander Health Partnership, Los Angeles, CA USA; 13 Center for Community Engagement, University of California at San Francisco, San Francisco, CA USA

**Keywords:** Cross-cultural communication, Black, Indigenous, People of Color (BIPOC), COVID-19, Health inequities, Community engagement

## Abstract

Lack of trust in biomedical research, government, and health care systems, especially among racial/ethnic minorities and under-resourced communities, is a longstanding issue rooted in social injustice. The COVID-19 pandemic has further highlighted existing health and socioeconomic inequities and increased the urgency for solutions to provide access to timely, culturally, and linguistically appropriate evidence-based information about COVID-19; and ultimately to promote vaccine uptake. California’s statewide alliance STOP COVID-19 CA (comprising eleven sites), leverages long standing community partnerships to better understand concerns, misinformation, and address racial/ethnic inequities in vaccine hesitancy and uptake. Using data from the California CEAL Communication Working Group, we demonstrate the wide range of strategies, communication methods, languages, and trusted messengers that have been effective in reaching diverse communities across the state. We also showcase challenges and lessons learned, such as the importance of including trusted community partners to share information or provide vaccines. These approaches, rooted in community engagement, are crucial for addressing inequities and responding to future public health emergencies.

**Supplementary Information:**

The online version contains supplementary material available at 10.1007/s10865-022-00284-8.

## Background

The COVID-19 pandemic has brought new attention to long-standing inequities faced by racial/ethnic minority populations and other underserved and socioeconomically disadvantaged groups. Not only have these communities borne the brunt of the pandemic in terms of negative health impacts (Iyanda et al., 2021; Nana-Sinkam et al., 2021; Riley et al., 2021), but complexities brought about by lack of access to essential medical resources and lack of trust of the scientific establishment due to institutional untrustworthiness and historical social injustices have made effective outreach and dissemination of evidence-based information about the pandemic difficult (Sy et al., 2020; Wilkins, 2018). Increased politicization of science and media has only added to the lack of trust in scientific information and resulted in uneven messaging and vaccination program implementation in different local communities. (Kates et al., 2021) Thus, there has been a need for community engagement and culturally and linguistically appropriate communication strategies to promote COVID-19 public health guidelines and ultimately promote vaccine uptake.

Black, Indigenous, Latino/x, and other communities of color have been disproportionately impacted by the COVID-19 pandemic, especially in terms of number of cases and fatalities (Boserup et al., 2020; Centers for Disease Control and Prevention [CDC], 2021; Iyanda et al., 2021; Riley et al., 2021). Factors rooted in inequities and social determinants of health and their intersection have played a large role in the heightened risk faced by these communities, among them lack of access to health care and stigma and stereotypes about poor hygiene and carrying disease. Overrepresentation in high-risk jobs (e.g., food and service industries), greater burden of chronic conditions associated with worse COVID-19 related outcomes, multigenerational households or overcrowded living conditions, immigration status, and barriers to healthcare access are just a few of these risk factors (CDC, 2021; Schulz et al., 2020; Upshaw et al., 2021).

In addition to experiencing higher risk and burden of the COVID-19 pandemic, these same communities have faced a variety of systemic challenges in accessing accurate and timely information about the course of the pandemic, including updates about vaccines and effective mitigation measures. Lack of access to linguistically and culturally appropriate materials (e.g., basic information about transmission, risk mitigation) have plagued public health responses. For example, during the early days of the pandemic, public information websites, hotlines, and online testing (and later vaccination) appointment systems were available primarily in English. The outreach methods associated with these resources often required reliable internet access, which represented a barrier for groups with limited technological access (e.g., not having broadband internet access or digital devices), those who had limited English proficiency, and people who had low levels of literacy. Many of the same racial and ethnic populations left out of English-only approaches have also lacked time away from work to receive testing or vaccinations, and they live in communities with long histories of disinvestment and transportation barriers (Moyce et al., 2020). Moreover, lack of trustworthy healthcare and government institutions stemming from historic institutional racism and harm created additional complexity for disseminating evidence-based information to communities. To overcomes these barriers, we need culturally appropriate communication strategies from trusted messengers (Sodeke, 2019; Wilkins, 2018). For instance, for many immigrant families, access to COVID-19 vaccines sometimes required disclosure of a social security number, which led to fears of deportation or additional unfavorable scrutiny of immigration status. In some communities, federal government agencies partnered with local agencies to identify and arrest undocumented immigrants [the 287(g) program], which suppressed the use of public programs by undocumented immigrants and even legal permanent residents and citizens (Wong, 2018). Communities that have been subject to medical mistreatment (e.g., Tuskegee experiment for the Black/African American community) had concerns that this was history repeating itself or that they would lack healthcare access after any potential vaccine side effects. Many community members also voiced skepticism about the outpouring of funds to promote the vaccines in contrast to the lack of support for basic needs and infrastructure that have plagued these communities well before the pandemic (e.g., support for food security, jobs, housing, or even other expensive medical care). In many of these instances, translation of materials without cultural or contextual adaptation was not sufficient to truly address valid community concerns and decades of strained community relationships due to social injustices and inequalities. These multitude of complex challenges spur the need to identify, develop, and implement culturally and linguistically responsive approaches, including community-engaged efforts, that are well suited to effectively communicate with diverse populations and collaborate with them, as partners, in the co-creation of culturally centered tools to promote COVID-19 prevention through vaccination.

These community-engaged approaches take a broader social justice perspective and are better able to overcome the aforementioned challenges associated with traditional institutional and research approaches. Rooted in principles of authentic partnership which include trust-building, power sharing, capacity development, co-learning and co-creation (Wallerstein & Duran, 2006), academic-community partnerships such as the ones presented in this paper have been identified as alternative strategies to decrease COVID-19 inequities. To highlight and advance equity-focused approaches, this paper will discuss important nuances around vaccine confidence and COVID-19 prevention and strategies implemented across the state, informed by existing community-engaged partnerships, to support racial/ethnic and other underserved communities disproportionately impacted by COVID-19. Using data from the California Alliance Communication Working Group, we demonstrate the wide range of strategies, communication methods, languages, and trusted messengers that have been effective in reaching diverse communities across the state. We also showcase challenges and lessons learned, and ways in which our work can be used for concurrent and future public health crises.

## Community strategies and community engagement for health equity formation of STOP COVID CA communications workgroup

**Fig. 1:**
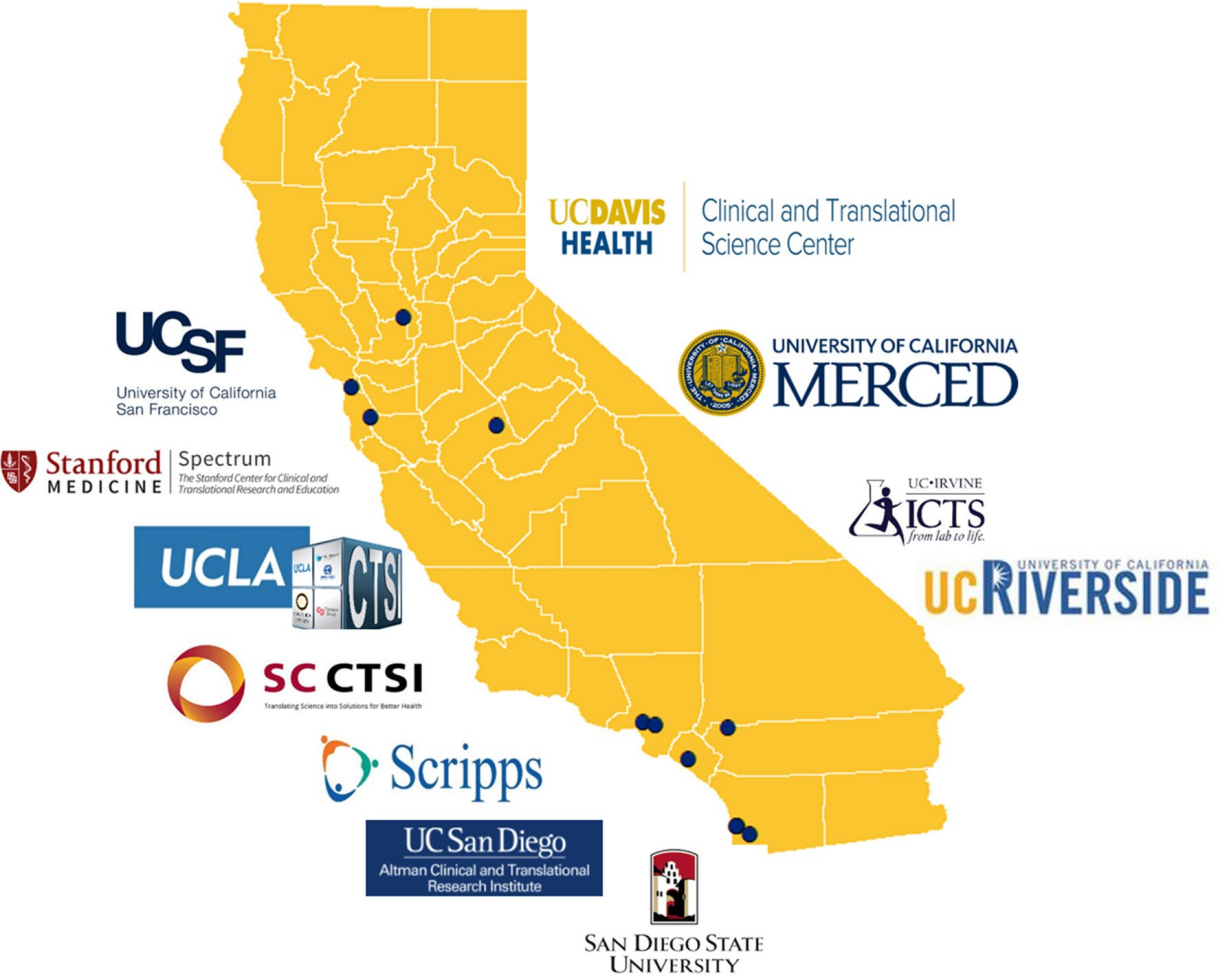
Map of the STOP COVID-19 CA team locations

As part of a National Institutes of Health (NIH)-funded statewide coalition formed in September 2020, the Share, Trust, Organize, Partner: the COVID-19 California Alliance (STOP COVID-19 CA) team comprises members from 11 academic sites and over 75 community partners across California (Fig. 1), hereafter referred to as the Alliance. To facilitate our work, we formed statewide working groups, one of which focused on COVID-19 communication and messaging (see Supplemental materials). Our biweekly Communications Workgroup (henceforth referred to as “Workgroup”) meetings were grounded in principles of community engagement, social justice, and cultural humility, meaning we kept open minds to learn from others and reflect upon personal biases. Our Workgroup members represent academic and community partners across urban and rural areas of the state working with diverse racial/ethnic groups (i.e., Latino/x, Black/African Americans, Native Hawaiian and Pacific Islanders [NH/PIs], Indigenous populations, Asian Americans) and people in high-risk occupations (e.g., farmworkers, community health workers). Each academic site had longstanding relationships with community partners from previous collaborations and the academic institutions had existing ties to each other as well. The community organizations include community advocates, community health workers, non-profit leaders, government officials (i.e., county and state public health departments), community-based media and immigrant advocacy organizations. For some of the community organizations, this initiative was the first time they had become involved with public health issues.

Our Workgroup meetings spent time discussing and identifying the populations that have been at highest risk for exposure to COVID-19 and/or high risk for severe disease, since terms like Black, Indigenous, and other People of Color (BIPOC) haven’t been sufficient for acknowledging all the groups at greatest risk of exposure. For example, our diverse statewide team includes and works with Native Hawaiians, Pacific Islanders and Asian Americans, groups consistently left out of conversations about COVID-19 despite high case rates or case fatality rates (Chang et al., 2020; Marcello et al., 2020; Yan et al., 2021). A feature of our work includes the recognition of local and cultural preferences an emphasis on tailoring materials using terminology that local community partners and populations prefer. For example, we discussed including the use of Latinx (gender neutral) versus Latino (traditional masculine term referring to groups with ancestry in Latin America, including South America), Hispanic (groups with ancestry in Spain), Chicano (traditional masculine term referring to groups with ancestry in Mexico, including Indigenous heritage), or other terms on communication materials designed for populations of Latin and South American descent. In addition to racial/ethnic backgrounds, our Workgroup paid special attention to the intersectionality of a multitude of social factors that expose many to greater risk for COVID-19, including occupation (e.g., essential workers, lack of paid sick leave), housing (e.g., multigenerational household, dense housing units), region (e.g., some rural areas lack testing or other medical care resources). Similarly, identifying high-risk occupations that often cross multiple racial/ethnic groups (e.g., farmworkers) helps with tailoring approaches to address specific barriers to receiving information or getting the vaccine.

We recognized that many communities have valid historical reasons to question the quality and provision of their healthcare, including vaccinations. By using culturally-centered approaches in our work, we aimed to understand those concerns (Wallerstein et al., 2019) and develop appropriate strategies to address them. This strategy goes beyond superficial tailoring (e.g., translating materials alone) and instead incorporating a deeper knowledge of the culture, values, and preferences, along with community access into all aspects of the process. One site specifically used a culturally grounded (Hecht & Krieger, 2006) and partnership-focused model to work with existing community partners to develop appropriate outreach strategies and educational materials better suited to addressing community member’s questions and concerns about the COVID-19 vaccines. Considering the impact of terminology on trust-building and implications of inadvertently blaming individuals or communities for voicing valid questions or concerns, the implications of language was of particular relevance for our group. Discussions in our meetings dove into the subtle nuances of terminology used to discuss COVID-19 mitigation strategies. For example, we discussed the implications of the term “vaccine hesitancy” instead of “vaccine confidence” or “vaccine deliberation.” The term “vaccine hesitancy” follows a deficit-based model that suggests the individual is at fault for unnecessary delay or avoidance of the vaccine while the latter two terms follow a strengths-based approach to suggest there are reasons for individuals to have confidence in the vaccines or that the individuals have agency in making their own decisions about vaccine uptake. We had similar discussions around the term “herd immunity,” which a workgroup member suggested should be replaced by “community immunity” to better illustrate the concept as well as the role of community members in helping to protect each other. This could also appeal to cultural values, such as familism in the Latinx community and collectivistic values of many Asian American, Native Hawaiian, and Pacific Islander communities.

## Communication strategies across communities and regions

Our Workgroup also regularly compared site- and population-specific communication strategies and messages in order to share resources and inform each other’s efforts (examples are described below and in Table 1). Project activities were approved under each site’s Institutional Review Board and were carried out by trained diverse (many bilingual) team members who reflected local communities. The Workgroup also includes several physicians and community leaders who shared the types of conversations and questions they had been having with patients and other community members in their geographic regions. Over time, our Workgroup has been able to track changes in the most common questions and most prevalent concerns as the pandemic evolved. For example, initial concerns focused on how quickly the vaccine was developed and potential side effects, which were answered through discussions about the lengthy research behind the vaccines and rigorous approval process, as well comparisons of the side effects to the flu and shingles vaccines. Current concerns include whether they are safe for children, which involves a discussion about the risks and benefits of the vaccine compared to the virus and possible long-term effects.

**Table 1:** Culturally Adapted Communication Strategies

Themes	CA Team Strategies	Description, adaptations and impact
Information gathering	Virtual town halls	●Specific events for marginalized communities (e.g., Veterans, LGBTQ, youth, etc.)
		●Features to enhance accessibility (e.g., closed captioning, live language translations, Facebook broadcasting)
		●Speakers included combination of academic or scientific experts along with trusted community leaders
		●Opportunity for bidirectional communication
	Focus groups	●Inclusive of communities often overlooked (e.g., Spanish-speaking Latino/x, Filipino/x, NH/PIs, refugees)
		●Also focused on key groups like youth and parents of young children
	Surveys	●Sampling methods and modes of distribution designed to include diverse and multilingual communities
	Meetings	●Inclusive of community members as partners in conversation and decision making, not just as recipients of information
Outreach	Phone	•Utilization of existing networks for other services (e.g., Census outreach) and subsequent creation of multi-language Wellness Phone Banking toolkit with regional resources and contact information
		•Free, confidential multi-lingual hotline to help with vaccine appointments
		•Allowing options to call or text message trusted messengers to make vaccine appointments
	In-person	•Leveraged trusted community resources to provide wraparound clinical and social services and an opportunity for (safe) face-to-face communications with trusted community leaders
		•Visible commitment to the community (e.g., presence at community events and in local venues)
Communication	Community health workers (CHWs)/promotoras and trusted messengers	●Partnerships with trusted messengers, especially bilingual/bicultural (e.g., faith leaders, CBO leaders, etc.)
		●Supported promotora-led programs (e.g., door-to-door outreach, community events)
		●Engaged with translators (including for Indigenous dialects) for short-term and long-term projects to be able to respond to rapidly changing guidance and recommendations. Able to translate into Indigenous dialects
		●Used familiar faces, languages, and cultural references from the community as foundation for trust and understanding
		●Used community artwork to promote the integration of traditional/cultural art mediums (e.g., Indigenous artwork)
		●Ensured that members from each community spearhead community activities (e.g., pop-up vaccination clinics) or artistic endeavors to increase community engagement and healing
		●Integrate creative ways of expressing COVID-19 information and experiences that will last longer in communities (e.g. mural development, art galleries, etc.)
	Multiple media channels	●Utilization of local ethnic media (e.g., radio stations, newspapers) to reach diverse communities
		●Use of varies methods (e.g., phone, social media, news, radio, flyers, door signs) to increase reach across different demographic groups and underserved communities
	Tailored messages and audience segmentation	●Developed tailored materials informed by understanding of the local and contextual needs and preferences of communities (e.g., radio vs internet, WhatsApp vs Facebook, farmworker vs urban communities), brought forth by community partners and information gathering phases
		●Understanding of preferences (e.g., trusted messengers, motivational messaging, etc.) across different age groups, racial/ethnic communities, regions, and other categories

## Methods for information gathering

Gathering the perspectives and concerns of the public was an important first step in developing effective communication materials to promote vaccine uptake. Especially at the start of our efforts, it was important to assess firsthand any COVID-19 concerns, barriers to accessing information or other care-related resources, and questions directly from community members. This was particularly relevant since the voices of many of these communities are often underrepresented in research. Given the constant and rapid changes in COVID-19 guidance, vaccine availability/eligibility, and level of community spread, it was important for Workgroup members to constantly update each other in virtual meetings and by email with new or ongoing community concerns as well as available resources and answers to questions. Since sites tended to work with different communities, age groups, and languages, sites were able to apply lessons from others sites, share virtual events, or re-purpose material and tailor it for new populations.

***Virtual town halls*.** Throughout the course of the pandemic, several STOP COVID-19 CA sites held virtual community town halls in multiple languages. These events provided an opportunity for community members to hear directly from local experts and get immediate answers to questions. Some sites, like Stanford, held monthly town halls in English and Spanish with active engagement through questions-and-answers with the audience; detailed analyses of the town halls will be published separately. In farmworker areas, call-in programs for monolingual Spanish speakers aired by the Radio Bilingüe radio station provided a virtual platform for discussion among a widely-dispersed population. In the spring of 2020, near the beginning of pandemic lockdowns, these town halls focused on disseminating evidence-based information about COVID-19 directly to communities and listening to concerns. Community questions initially focused on safety guidelines and proper use of personal protection equipment (PPE), vaccine clinical trials, and more recently vaccination campaigns and hopeful recovery. Over time, these town halls evolved to have fewer formal presentations and instead serve as fora open conversations with experts and community leaders on topics that range from vaccine safety to community concerns (such as the pandemic’s impact on mental health). To answer as many questions as possible, Stanford’s town halls had panelists provide in-depth responses to key audience questions, while other team members behind-the-scenes simultaneously answered additional questions submitted through the platform. The teams in Los Angeles held virtual sessions with community groups, ethnic media, schools, and faith-based organizations. These were unique opportunities to directly pose questions to experts, as well as gain a sense of the types of information and priorities needed for the development of additional dissemination materials. These sessions often paired community leaders with scientific experts in order to leverage community credibility as well as subject matter expertise – responses were even more positive when scientific/clinical experts were fellow community members. Community members were compensated for their time through financial means or other resources. For example, a collaboration among the Indian Health Council, Inc., University of California, Riverside, and Scripps featured an Indigenous/Native American physician and physician-in-training to answer questions about the COVID-19 vaccines and variants. Another virtual town hall featured the National Basketball Association (NBA)’s medical director (a Black physician) describing lessons from his successful “bubble” that allowed the NBA to finish their 2020 season; it was so well-received that the session went well past its scheduled end. Bilingual/bicultural presenters (especially clinicians and scientists) have received positive community feedback from audience members who feel they can trust the information more because they are receiving it in their native language.

### Focus groups

Several sites have been conducting paid virtual focus groups in communities most impacted by COVID-19 and/or with potentially unique concerns, such as racial/ethnic groups (e.g., Black/African Americans, Latino/x, Filipino/x, Native Hawaiian and Pacific Islanders) or age groups (e.g., youth), or other characteristics (e.g., parents). Findings from focus groups were used to guide outreach strategies and inform development of vaccine-related education and promotion materials, especially since vaccine-eligibility and vaccination rates changed over time. Focus group guides were specific to each site’s unique population and needs. Learnings were shared during the Workgroup meetings. For example, Workgroup members from San Francisco found that youth (ages 18–25) relied almost exclusively on social media, where there is a great deal of misinformation, for information about the pandemic. Online focus groups with Southern California parent-adolescent dyads elicited parent and adolescent concerns and questions about vaccination. These focus groups were conducted in anticipation of future emergency authorization for COVID-19 vaccination for adolescents (and subsequently for children under the age of 12). Results indicated low vaccine confidence and high COVID-19 risk complacency for adolescents as emergent themes among parents, although they would be motivated by explicit healthcare provider recommendations, school or travel mandates, protecting their child’s/family’s health, the ability to resume social activities, and receiving updated vaccine information.

Focus group findings across our sites were discussed in our statewide meetings to help inform communication strategies and priorities across sites. Sites in both Northern and Southern California had focus groups focusing on the needs of specific racial/ethnic communities (e.g., Indigenous, Chinese, Filipino/x, Latino/x) and were often facilitated by members of those respective communities. This cultural congruence helped to ensure that focus group participants felt safe to discuss their needs and concerns surrounding COVID-19 and vaccines, including concerns about immigration status, potential side effects, discriminatory treatment, and access barriers. Some sites, like the Inland Empire team, trained community members to facilitate focus groups, allowing for both information-gathering and capacity building.

### Surveys

Sites across the state disseminated electronic and paper surveys to gauge understanding and practice of COVID-19 safety guidelines, knowledge about the vaccines, and intention to vaccinate. Respondents received financial compensation (e.g., gift card) or other free resources (e.g., PPE). Survey dissemination considered different population needs, trust, and tailored advertising in order to be truly reflective of communities particularly impacted by COVID-19. In the Inland Empire region, survey information was distributed by trusted members of communities of interest. Such communities included migrant farm workers, undocumented immigrants, and non-English speakers. Survey information was also spread through trusted cultural institution such as churches, Black barbershops and the *Riverside – San Bernardino County Indian Health, Inc.* (RSBCIHI), a Native American healthcare organization serving Inland Empire tribal members. Detailed analyses of the surveys will be published separately.

### Meetings

Regular meetings and discussions with key partners (e.g., promotoras, community advisory groups, faith leaders) also helped to keep the teams at each site updated with current community concerns and questions. Although there are frequent scientific updates regarding COVID-19 or the vaccines, understanding what communities are actually hearing or most concerned about was helpful in prioritizing efforts, particularly when there were discrepancies in the emphasis of certain information between scientific communities nationwide and local community partners and populations we were working with. For example, by the late spring/early summer 2021, based on emerging scientific recommendations, state guidelines no longer required mask-wearing for the fully vaccinated. We reassured community members that they could continue doing what made them comfortable, especially for the immunocompromised, their caregivers, or families with young children. After the delta variant brought the return of local mask mandates, some community members expressed relief that they hadn’t stopped wearing masks.

## Outreach strategies

Contrary to the “low touch” (English-only online platforms) but easy-to-scale methods to provide COVID-19 information and vaccine appointments across the state, our respective sites developed “high-touch” outreach strategies that involved more time (i.e., training staff and volunteers) but were more tailored to local community needs and met people where they live, work, and worship. During this pandemic, many of the community members we worked with have lacked paid time off, lost incomes, and faced increased responsibilities to care for elders and homeschool children (sometimes without broadband internet access). Therefore, it was important that we bring information to people rather than expect them to find a way to get online and navigate complex English-only websites. These high-touch outreach strategies required more time and personnel investment, but our workgroup members agreed that for many communities experiencing the digital divide or are hesitant to interact with cultural outgroups (particularly the elderly and those with limited English ability). The information gathering strategies described above helped to inform the outreach strategies described below. Sites like UCSF and partnering community-based organizations (CBOs) prepared policy briefs that described recommended strategies for rapid dissemination of information to communities; these briefs were shared with local public health and community leaders to inform municipal and community-based vaccination efforts. Similarly, the SoCal (Southern California) Pacific Islander Task Force helped a local government agency to be more inclusive of Native Hawaiians and Pacific Islanders through the use of more representative graphics and multi-language content. Multiple sites reported successful turnouts at community vaccination events, with anecdotal comments from community members who trusted these community leaders and events more than other people and vaccination sites. For example, a small community health fair in San Diego in one of the lowest-income areas of the county had a few days’ notice that they were approved to provide vaccines in early March 2021; an overwhelming number of community members lined up long before the fair even opened, resulting in the administration of 1,087 vaccine doses. There are similar anecdotes from community members who declined the vaccine until the opportunity arose with trusted community leaders at familiar community sites.

### Phone outreach

For communities that culturally have preferred face-to-face or more personal interactions via the phone, like Native Hawaiian and Pacific Islanders, the inability to talk in person was challenging. In Southern California, previous phone banking efforts from voter registration and Census outreach were used to launch “Wellness Phone Banking” by Native Hawaiian and Pacific Islander community-based and faith-based organizations to check in with their constituents to determine their needs, e.g., prescriptions, food delivery, and other resources. This type of personal contact from trusted community members was helpful for both gathering information and sharing resources. The inclusion of additional community resources also serves to emphasize social determinants of health, which were exacerbated during the pandemic for many communities of color. In the more rural Central Valley, a free and confidential STOP COVID-19 San Joaquin Valley call line (in English and Spanish) was set up to allow UC Merced students to help community members make vaccination appointments. In San Diego, community leaders relied on phone calls and text messages to set up vaccine appointments, which meant they needed to manually enter information into the local immunization registry. However, this level of “high-touch” effort was necessary because community members otherwise faced barriers with vaccine appointments that required navigating complex English-only websites or patient portals.

### In-person outreach events

Tabling (following safety guidelines) at community health fairs or other community events has been an effective strategy during a time when many community members are seeking a personal connection. These events provided an opportunity to learn more about current individual concerns, share updated scientific information, and connect people to needed resources. More importantly, these opportunities placed COVID-19 information as one of several key resources (i.e., wraparound services like free groceries and free health screenings) that community members needed. Multiple sites shared examples of using food distribution sites to disseminate information about the COVID-19 vaccines, nearby vaccination sites, or generate information about mitigation strategies. In San Diego, the table with COVID-19 vaccine information also offered free PPE kits (including masks and bottles of hand sanitizer) to demonstrate that vaccines are part of an overall pandemic mitigation plan out of concern for the general safety of community members. This form of in-person interaction allowed for sharing of printed materials (e.g., testing and vaccination site locations) for community members with limited or no internet access.

## Communication strategies

The foundation of our communication strategies has been its bidirectional nature where the speakers or information sources and key audiences are able to meaningfully communicate with each other across platforms and language. An overwhelming volume of COVID-19 information exists and continues to be rapidly disseminated. Yet, there has been little space for processing and having dialogues with the most impacted communities that reflects understanding, caring, and creates a safe space for exploring institutional mistrust and other concerns (e.g., around the vaccine). We turn to trusted messengers and community leaders, especially bilingual and/or bicultural, who are able to share information with key audiences in culturally relevant terms and provide space and time for active dialogues where participants can ask questions.

### Community health workers/promotoras and other trusted messengers

The key to the information gathering and outreach strategies described above has been the use of trusted messengers. Trusted messengers include community health workers, faith leaders, and leaders from CBOs who have long-term histories of providing services to communities and have shown commitment to the community despite having minimal financial resources. Some volunteer and provide their time outside of regular jobs and caregiving responsibilities. As community insiders, they bring a level of credibility, understanding, and familiarity to interactions with community members. As seen throughout the pandemic, disseminating biomedical information through institutional channels (i.e., federal agencies, public health departments, universities, mainstream media, etc.) has not effectively reached marginalized groups. Being able to provide information to communities of color and disenfranchised communities in culturally and linguistically relevant ways is crucial to successful public health messaging. Therefore, our group has attended to the unique needs, characteristics and preferences that community members may have in receiving information.

Using a more grassroots approach, our work has allowed us to co-design dissemination of evidence-based information and engage groups that have not been a major focus of local, regional, or national efforts. Another important strategic component, based on insights from social psychology and marketing research, was to design public service announcements (PSAs) with special attention to effectively conveying “authenticity” to overcome audiences’ distrust and disregard of standardized messages from “outsiders” as evidenced by style of communication and specific terminology used. Various stations complemented these PSAs with call-in public affairs program segments (Línea Abierta and Hora Mixteca) where on-air hosts invited calls from listeners to share their concerns and questions and get answers from on-air hosts and guests.

### Multiple media channels

The nature of the pandemic created an overreliance on digital communications, but this was not sufficient to reach the most at-risk populations. Therefore, the different sites took creative approaches to reach key populations. For example, to reach farmworkers who lacked internet access, Workgroup members from UC Davis developed a strategic communication framework to guide its collaboration with Radio Bilingüe, the national Latino/x public radio network. The framework focused on persuasive communication, not simply “information dissemination” in order to promote vaccine uptake. Within this framework, social media-ready short films and videos contain culturally appropriate imagery, feature trusted messengers, and are in needed languages. UC Riverside team members are using an art-based approach to convey messaging surrounding COVID-19, ranging from community testimonials to vaccine information. They work with local artists to develop murals, art exhibition, art contests and open microphone nights to boost COVID-19 related information and better capture community narratives surrounding COVID-19. This approach is meant to foster community healing through creative engagement, break down linguistic barriers, and provide long-lasting symbols of COVID-19’s impact and the ways in which individuals can promote health and safety. The UCLA team participated in interviews for local Spanish-language TV, radio, newspapers, Black/African American newspapers in addition to English-language programs.

### Tailored messages and audience segmentation

Although the pandemic is often characterized in mainstream media using quantitative measures (e.g., numbers of infections, hospitalizations, or death), personal narratives often resonate more than statistics, especially when numbers are in the millions. (Olson, 2015). For example, conversations about the decades of scientific effort behind the vaccines need to be accompanied by personalizing the scientists and also sharing personal testimonials about the vaccine. Many of the communities hardest hit by the pandemic rely on oral tradition and storytelling, so it also makes more sense to follow more traditional forms of communication when reaching out to these groups. For the UC Davis team, an important element in their strategy was to not simply rely on a radio station’s decades-long track record as a “trusted voice” on health issues but to target sub-populations within the station’s Spanish-dominant audience (e.g., youth, middle-aged couples, indigenous farmworkers) by placing PSAs in appropriate program segments to reach these various audience segments. Initial message themes were assessed in a focus group with farmworkers and ongoing community conversations by station staff at a range of events, including the local celebration of a Mixtec migrant-sending community’s saint’s day (San Juan Bautista). For the UCSF team, that meant pivoting to youth leaders to reach youth about the vaccines. As a workgroup, we learned from each other’s outreach and strategies to inform our own work, all which continued to evolve with the pandemic.

## Challenges and future considerations

Several of the challenges with COVID-19 communications reflected the difficulty with keeping up with a rapidly changing global pandemic, as well as systemic issues that predated the pandemic. These conditions, along with the rampant, far-reaching misinformation and disinformation campaigns, continue to make it difficult to keep ourselves and trusted messengers updated with the latest science in order to be proactive with anticipating needs and upcoming priorities without adding to confusion.

## Lacking infrastructure for inter-agency information sharing

Lack of a consistent infrastructure for sharing information meant that information from federal, state, and county agencies did not always match or allow space for direct feedback. Although the implementation of any guidelines may need local tailoring, the lack of uniform messaging (e.g., masking guidelines about who should wear masks and when) have led community members to question the true motivation behind the messaging and/or not understand the message. The unidirectional flow of information has made it difficult to respond rapidly to community needs and concerns. In order to better coordinate collective efforts and guidance, it would be helpful for various levels of key agencies and organizations (i.e., local, state, federal) to proactively offer opportunities for bi-directional information/feedback, and advance notice of communications. This was a key strength of our Workgroup: we were able to incorporate feedback and information across sites to adjust strategies, including direct feedback from community partners most familiar with local needs and context.

## Difficulty in communicating rapidly changing information

The constant changes in COVID-19 guidelines (e.g., effectiveness of masks, social distancing guidelines, whether younger people are at risk for COVID-19) have added to confusion and mistrust. Moreover, the sheer volume of communication channels now available, from mainstream media to social media, in addition to the wide range of language needs, makes it difficult to identify current, factual, and understandable information. These challenges have made it difficult to be proactive and anticipate upcoming messaging priorities. In particular, developing persuasive, effective, and culturally and linguistically relevant messaging takes time. Universal or population-wide messages can rapidly become outdated and were generally less effective in reaching diverse communities. Thus, there is a need to adapt messaging as new information becomes available, while being proactive in anticipating future messaging. For example, our teams are currently developing materials to prepare families for subsequent phases of vaccination program rollout for younger children.

## Initial lack of support and capacity for trusted messengers

Initially, COVID-19 information was only shared through institutional and mainstream channels, with a delayed recognition of the need for trusted messengers to effectively reach marginalized communities (e.g., low-income, racial/ethnic minorities, undocumented, immigrant). When the focus shifted to having trusted messengers deliver COVID-19 information, there was insufficient time to build capacity (e.g., provide paid training about COVID-19, increase staffing). There was also an increased burden placed upon trusted messengers, including community health workers (CHWs), who were in extremely high demand due to their expertise and credibility, but were provided with limited to no resources (i.e., compensation, mental health and wellness support, etc.). This network has provided training and compensation for CHWs, but has faced bureaucratic challenges getting the funds to the CHWs in a timely manner. This raises the need to increase awareness and accountability among academic institutions and research funders for establishing fair and timely support for community partners and valuing their expertise and support in sponsored research (Black et al., 2013). Moreover, the uncertain timeline of this ongoing pandemic has also created challenges for addressing the stress and mental health faced by CHWs. Teams from UCLA have co-created restorative healing circles and opportunities for partners to process the pandemic. All sites were offered opportunities to host local healing circles. Additionally, other sites have also incorporated regular check-ins and opportunities to discuss pandemic-related stress, burn-out, and share coping strategies. A distinctive challenge and opportunity in farmworker communities was the need to work with agricultural employers and farm labor contractors to encourage vaccination among their workers and go further “reaching out with a helping hand” to organize vaccination events in the workplace or living quarters. Related to this has been the extraordinary (but predictable) delay in translating resources into other languages or acknowledging cultural nuances across communities.

## Challenges in combatting misinformation

For community members with limited English proficiency and/or limited internet access, resources—for everything from preventing COVID-19 to testing or vaccine sites—have been out of reach. Furthermore, these resources often use excessive jargon, overly complex sentence structures, or provide infeasible recommendations (i.e., telling essential workers to stay home when sick or multigenerational households to self-isolate). Low health literacy is a common problem even among the most highly educated, and is compounded by language barriers and the digital divide. Thus, these communities have experienced real barriers to timely information. Unfortunately, misinformation about COVID-19 and the vaccines has been plentiful in many languages and across platforms more easily accessible to community members, creating an urgency for official public health campaigns to match readily available misinformation with factual, culturally and linguistically appropriate messaging.

The spread of misinformation via social media (i.e., Twitter, Facebook, Instagram, WhatsApp, etc.) is rampant and has been particularly effective in dissuading communities who may have low levels of digital literacy. Gaps in digital literacy—or one’s ability to find, evaluate and critically assess information shared digitally – disproportionally impact BIPOC and communities with low levels of education. Building capacity to identify inaccurate or misleading information is especially crucial as the pandemic progresses, given that misinformation continues to change frequently. Creating and distributing digital literacy resources via schools, local municipalities, libraries, and community-based groups may help mitigate the effects of misinformation among under-resourced communities. Providing these resources to adults is also crucial, ideally paired with tools (e.g., broadband internet access, digital devices) to bridge the ongoing digital divide. Building digital literacy can also serve as asset in responding to current and future public health crisis.

## Lessons for the future

There is little doubt that the United States (and the world) needs to prepare for future pandemics and other public health crises. We posit that there are existing health crises, from food insecurity to chronic disease such as diabetes, that also disproportionately affect the same communities impacted by COVID-19 but have lacked long-term solutions. Existing efforts that address these health crises also fail to actively engage impacted communities as partners and acknowledge structural injustices that further facilitate health disparities. Current public health crises will most likely be amplified in the coming decades, with threats of climate change and global injustice perpetuating health inequities. Climate change and its disproportionate impact on low-income communities of color, for example, is likely to require community-engaged attention and intervention to be effectively and equitably addressed. The lessons learned from the Workgroup (see Table 2) could serve as a guidepost for future public health emergencies and for addressing other long standing health inequities.

**Table 2:** Recommendations for future coalitions

Recommendations	Known Challenges and Key Strategies
Increase community and academic capacity to enhance community-academic partnerships	*Known Challenges*
	•Community organizations have limited resources and are often staffed by volunteers, which can make it difficult to fully participate in academic projects
	•Many project roles have academic-focused qualifications (e.g., college degree) while common community skillsets (e.g., language skills, knowledge of community cultural preferences, networks of community organizations or leaders) are not valued to the same extent
	•Traditional research methods (e.g., formal surveys) don’t always work well with communities underrepresented in research
	•There is often a mismatch in preferred methods of communication dissemination among academic and community organizations (e.g., academic journals vs policy briefs, news articles, etc.)
	*Key Strategies*
	•Provide resources for community organizations to support long-term (not project-dependent) staffing and enhance staff skills
	•Reduce bureaucratic obstacles to obtaining funding (e.g., rules about organizational status, volume of paperwork) and later to support sub-awards to reduce administrative burden on community partners
	•Increase flexibility with staffing to allow for staff skills that reflect actual community needs (e.g., skills, languages) and allow for participation in projects (e.g., evening or weekend meetings, reduced use of jargon)
	•Recognize the value of qualitative research methods and other non-western ways of knowing (e.g., oral story telling), which may provide additional context to understand needs of underrepresented populations in research
	•Encourage expansion of preferred methods of communication to increase broader dissemination of information (e.g., blogs, podcasts, radio, policy briefs)
Trust: Invest in trusted messengers and increase the trustworthiness of academic institutions	*Known Challenges*
	•Community partners are often overlooked or included much later in projects, after funding or study design decisions have already been made
	•Community partners are often unfunded or underfunded for their time and contributions
	•Overextending and overreliance on the same trusted messengers can lead to burnout
	•Translations of materials to other languages is de-prioritized or not done at all, leading to misunderstandings and linguistic isolation. Other times, the effort it sometimes superficial and in the absence of other cultural considerations, deters effective knowledge transfer
	*Key Strategies*
	•Build community relationships over time, ideally without the pressure of academic deadlines or pending grants, to allow for more meaningful long-term collaboration and shared decision making
	•Invest in paid opportunities for community partners to aid in knowledge transfer, co-development of information, and signal valuing time and skills of community partners in the scientific process
	•Increase academic staff diversity (especially bilingual, bicultural individuals) and train existing staff to better understand and respond to community needs; it is everyone’s responsibility to be trustworthy
Consider long-term cross-site partnerships	*Known Challenges*
	•Organizations are accustomed to working in siloes and unidirectional communication, whether due to funding, academic credit, different goals, or other reasons
	•Different messages coming from multiple agencies, especially when information rapidly changes, can contribute to mis-information and fuel overall public mistrust
	*Key Strategies*
	•Build infrastructure for inter-agency information sharing to encourage bi-directional communication across organizations in different sectors
	•Encourage organizations to find shared or mutually beneficial goals that can lead to coordinated efforts; this can help avoid redundancy and mixed messages to the public
	•Leverage partnerships developed during the COVID-19 response to address other public health issues of concern

## Focus on community partnerships and capacity development

Recognition of the value of community engagement and authentic bi-directional partnership is paramount. Investment in our communities from the ground up and dispelling preconceived ideas about community members by agencies and corporations would be helpful. This means making long-term investments in these relationships through financial support, capacity development, and general support – long before there is a need or another emergency. Financial investments, such as the National Institutes of Health (NIH) Community Engagement Alliance (CEAL), which funded our statewide efforts, are crucial in providing coverage and resources to rapidly implement projects. This funding, coupled with the robust, longstanding partnerships between academic institutions and community partners, allowed the Alliance to quickly launch and amplify community voices and efforts as the state began to promote vaccinations across California, particularly in communities hardest hit by the pandemic. Our experience suggests that additional funding outside of emergency responses will likely help academic and community organizations to develop new partnerships and foster trust and collaboration with other communities and populations not currently represented in large federally funded projects. This will also signal to community members that it is worthwhile to participate in these efforts and invest on their end, despite long histories of advocacy with minimal success.

Capacity development is also a major component of fostering long-term change, increasing access to information, and creating infrastructure to support partnerships and bidirectional communication to respond to future challenges. A large portion of our work has centered not only on development of culturally-centered information, dissemination and engaging community members, but also on providing all partners with tools to continue this work for years to come. For example, the creation of community toolkits that incorporate techniques for community engagement, health communications and healthcare resources ensure that community partners and organizations can support one another and extend their knowledge beyond the groups involved in our project. Toolkits are designed to be easily comprehensible, widely accessible and multilingual, reducing the use for medical jargon and inaccessible language. Similarly, developing websites, physical resource kits, social media campaigns, videos, and small guides to distribute among communities serve to both disseminate health information while providing individuals with additional skills in health promotion. These resources can also serve as a training resource for new members of community organizations and others involved in similar projects in the future. Within our own teams, community partners indicated that such resources also aid in language revitalization and cultural inclusivity, such as translating slogans and information into Cahuilla, Mixtec, Zapotec, Triqui, Hmong, Samoan, Tongan, Native Hawaiian and other languages often not included in translation and adaptation efforts.

To build the capacity of academics, our integrated Workgroup allows for knowledge exchange that allows for additional understanding of local needs and recognition of community-identified concerns and solutions (vs researcher-imposed plans). In addition, this collaboration has renewed advocacy within our respective academic institutions for increased support for community-engaged research and changes at the institutional level to decrease barriers for future partners to meaningfully engage with academic institutions (e.g., IRB, burden of paperwork required for subcontracts with community organizations) and also increase the visibility of community expertise and work. Thus, there is much room for improvement for institutions and academic partners to actively strive to increase trustworthiness, reduce the administrative burden on community partners, incentivize community-institutional partnerships, and ensure our ability to respond in real-time to public health emergencies.

## Invest in trusted messengers and increase institutional trustworthiness

This is a long-term investment in capacity building and support for trusted messengers, especially community health workers, must also be balanced with the acknowledgement of this limited human resource. Instead of being completely reliant on the same small group to serve as trusted messengers for communities, we must create long-lasting infrastructure to foster and expand community-based health approaches that feature empathy, relationship-building, and bi-directional communication. Trusted messengers don’t have to be limited to community leaders – scientists, physicians, and officials can learn to become trusted messengers and increase the trustworthiness of their respective institutions. This would both expand the pool of trusted messengers and support future outreach efforts, but also prevent burnout that many trusted messengers may have experienced throughout the COVID-19 pandemic. Ensuring that experts in other fields and community leaders are trained to understand the impact of the social determinants of health as well as cultural nuances can help foster a greater sense of trust and community receptivity. This is not a replacement for working with communities; through training and bi-directional partnerships with impacted communities, experts and prominent community members can obtain a deeper understanding of the ways in which health behaviors are created and promoted among historically marginalized communities as a result of historic trauma and institutional oppression. These efforts must also be tempered with the knowledge that no single person or organization can represent an entire community; there is great diversity within groups (i.e., different dialects or countries of origin) and that there should be a vetting process to ensure that trusted messengers continue to provide factual information and don’t use their credentials or reputation to create misinformation. It is also crucial to recognize the diversity within racial/ethnic neighborhoods, understanding that no single organization’s “trusted voice” can effectively reach everyone within communities.

## Consider long-term cross-site partnerships

Collaborations across public health agencies, community organizations, policymakers, academics, and others are helpful for amplifying each other’s work and creating more unified messaging. Local, statewide, and national alliances such as those involved in CEAL efforts, including our own Workgroup, offer opportunities for cross-site learning, networking for those working with similar populations or using similar strategies, and serve to amplify messaging and co-develop intervention strategies. These coordinated approaches could be used to help prevent and combat chronic disease or address climate change, issues which again help to address health equity. Moreover, such efforts would strengthen information credibility and prepare local, regional and larger-scale groups to answer questions and concerns that community members may have regarding new policies, health information, etc.

## Conclusion

The Workgroup’s wide range and iterative strategies illustrate the possibilities for more effective messaging better suited for multicultural populations and can serve as a model for other public health emergency responses. We have demonstrated how integration of community engaged, culturally and linguistically appropriate strategies within all aspects of our work has allowed us to develop messaging and effectively partner with diverse communities that have been the hardest-hit by the pandemic. These strategies also allow us to overcome institutional limitations and foster trust and inclusivity and support long-term health partnerships to building public health infrastructure rooted in grassroots and community-based approaches. Specifically, our efforts helped to resolve uncertainty about the COVID-19 vaccines, provided diverse communities with increased access to the vaccine from trusted providers and helped community organizations and the populations they serve obtain other needed resources during this challenging time. COVID-19 is just one of many urgent health concerns that disproportionately affect low-income populations and communities of color across geographic regions. We need to use this opportunity to continue building trustworthy relationships that can be leveraged in future health equity efforts and investment (from the local, state, and national levels) in academic-community partnerships, as well as in local communities and leaders in order to have a more holistic perspective of the root of the problems, as well as potential solutions. This Alliance has shown what is possible with financial support and capacity development. We strive to be a model for future and ongoing public health responses to promote health equity approaches.

## Supplementary Information

Below is the link to the electronic supplementary material.

Supplementary file1 (DOCX 15 kb)
