# Supplementary material for: Addressing racial/ethnic inequities in vaccine hesitancy and uptake: lessons learned from the California alliance against COVID-19
Source: J Behav Med. 2022 Jan 22;46(1-2):153–66. doi: 10.1007/s10865-022-00284-8 (PMC8783654; doi:10.1007/s10865-022-00284-8)
Supplement: Supplementary file 1 — Supplementary file1 (DOCX 15 kb) [file 10865_2022_284_MOESM1_ESM.docx]

Supplemental Materials

**Supplemental Materials Table 1**

*Contributors to the California Communications Workgroup*

| **Institution** | **Contributors** |  |
| --- | --- | --- |
| Global Action Research Center | Paul Watson |  |
| Multicultural Health Foundation | Robert Gillespie |  |
| Pacific Islander Health Partnership | Audrey Kawaiopua Alo |  |
| San Diego State University | Claudia Carrizosa |  |
| Scripps | Mona AuYoung, Olivia Ramos Cruz |  |
| Stanford School of Medicine | Wei-ting Chen, Patricia Rodriguez Espinosa |  |
| United Way Merced | Claudia Corchado |  |
| University of California, Davis | Sergio Aguilar Gaxiola, Luis Carvajal Carmona, Melisa Price |  |
| University of California, Irvine | Suellen Hopfer |  |
| University of California, Los Angeles | Paris Adkins-Jackson, Etsemaye Agonafer, Arleen Brown, Juan Barron, Savanna Carson, Alejandra Casillas, Sarmen Hakopian, Gloria Kim, Ejiro Ntekume, Stefanie Vassar |  |
| University of California, Merced | Maria-Elena De Trinidad Young |  |
| University of California, Riverside | Shaleta Smith, Preeti Juturu, Ann Cheney |  |
| University of California, San Francisco  University of Southern California  Werner Kohnstamm Family Giving Fund | Lydia Leung, Roberto Vargas  Allison Orechwa  Ed Kissam |  |
